# Multiple linear epitopes (B-cell, CTL and Th) of JEV expressed in recombinant MVA as multiple epitope vaccine induces a protective immune response

**DOI:** 10.1186/1743-422X-9-204

**Published:** 2012-09-17

**Authors:** Fengjuan Wang, Xiuli Feng, Qisheng Zheng, Hongyan Hou, Ruibing Cao, Bin Zhou, Qingtao Liu, Xiaodong Liu, Ran Pang, Jin Zhao, Wenlei Deng, Puyan Chen

**Affiliations:** 1 Key Laboratory of Animal Diseases Diagnosis and Immunology, Ministry of Agriculture, Nanjing Agricultural University, Nanjing, 210095, China; 2 National Veterinary Biological Medicine Engineering Research Center, Nanjing, 210014, People's Republic of China; 3 Veterinary Office, Gansu Agriculture and Animal Husbandry Department, Lanzhou, 730000, People's Republic of China; 4 Veterinary Medicine, Yangzhou University, Yangzhou, 225009, China

**Keywords:** Japanese encephalitis virus, rMVA-mep, Immune response, Protection response

## Abstract

Epitope-based vaccination might play an important role in the protective immunity against Japanese encephalitis virus (JEV) infection. The purpose of the study is to evaluate the immune characteristics of recombinant MVA carrying multi-epitope gene of JEV (rMVA-mep). The synthetic gene containing critical epitopes (B-cell, CTL and Th) of JEV was cloned into the eukaryotic expression vector pGEM-K1L, and the rMVA-mep was prepared. BALB/c mice were immunized with different dosages of purified rMVA-mep and the immune responses were determined in the form of protective response against JEV, antibodies titers (IgG1 and IgG2a), spleen cell lymphocyte proliferation, and the levels of interferon-γ and interleukin-4 cytokines. The results showed that live rMVA-mep elicited strongly immune responses in dose-dependent manner, and the highest level of immune responses was observed from the groups immunized with 10^7^ TCID_50_ rMVA-mep among the experimental three concentrations. There were almost no difference of cytokines and neutralizing antibody titers among 10^7^ TCID_50_ rMVA-mep, recombinant ED3 and inactivated JEV vaccine. It was noteworthy that rMVA-mep vaccination potentiates the Th1 and Th2-type immune responses in dose-dependent manner, and was sufficient to protect the mice survival against lethal JEV challenge. These findings demonstrated that rMVA-mep can produce adequate humoral and cellular immune responses, and protection in mice, which suggested that rMVA-mep might be an attractive candidate vaccine for preventing JEV infection.

## Introduction

The Japanese encephalitis virus (JEV), which belongs to the family Flaviviridae, infects the human central nervous system [1,2]. The virus has a zoonotic transmission cycle between birds and mosquitoes, in which swine serves as an intermediate amplifier hosts and JEV can spread from swine to humans via mosquito bites [3-5]. Thus the vaccination of swine against JEV should contribute to minimize the occurrence of JE epidemics in humans [6].

Both inactivated and live-attenuated vaccines have been widely used in many Asian countries. Inactivated mouse brain vaccines are considered first generated, inactivated cell culture vaccines and live SA14-14-2 are considered second generation vaccines against JEV [7], which play vital roles on effectively decreased the morbidity of Japanese encephalitis. During the vaccination with both inactivated and live-attenuated JEV vaccines, there are various disadvantageous conditions, including poor availability, high production costs, poor long-term immunity, and the possibility of allergic reactions [8]. These problems may be solved by using epitope-based vaccines containing selected protective epitopes, appropriately presented, which are capable of stimulating effective B cell, T cell, and cytotoxic immune responses while avoiding the induction of undesirable side-effects [9].

Judging from animal experiments, the protective immune response against JEV infection arises via both humoral and cellular immunity. Passive immunization with monoclonal antibodies specific for the JEV E protein has been shown to protect animals from lethal JEV challenge [10,11] and the adoptive transfer of JEV-specific cytotoxic T lymphocytes (CTLs), which confer protection against lethal challenge in mice [12]. Likewise, the envelope (E) protein of JEV was reported to be a strong immunogen for the production of neutralizing antibodies and CTLs [13]. Many vital epitopes identified in the JEV E protein [14-16] are effective in inducing protective immunity against JEV. Moreover, it has been reported that the design and delivery of epitope-based vaccines have the potential to be a novel vaccine against JEV [17-19].

In recent years, modified vaccinia virus Ankara has showed great promise as vectors for recombinant vaccine development [20-24]. Despite its replication deficiency in human and most mammalian cells, MVA provides high-level gene expression and has been proven to be immunogenic when delivering heterologous antigens in animals and humans [25-27]. MVA vector vaccines induce significant levels of humoral and cellular immune responses to vaccine antigens and were found to be less affected by preexisting vaccinia virus-specific immunity when compared to replication-competent vaccinia virus vectors [28]. In this study, we first constructed the recombinant MVA by synthesizing multi-epitope gene from JEV envelope protein, which combined six B-cell epitopes (amino acid residues 75–92, 149–163, 258–285, 356–362, 373–399 and 397–403) and a CTL epitope (amino acid residues 60–68) and a Th epitope (amino acid residues 436–445) [29], and evaluated the abilities to induce immune responses in mice and protective efficacy against JEV challenge.

## Materials and methods

### Cells and mice

Baby hamster kidney (BHK-21) cell (ATCC CCL-10) and rabbit kidney cell (RK-13) cell (ATCC CCL-37) growed in Dubach’s modified Eagle’s medium (DMEM) supplemented with 10% heat-inactivated fetal bovine serum (FBS), 100 μg/mL of streptomycin, and 100 μg/mL of penicillin.

Female BALB/c mice (4–6 weeks old) were purchased from the Experimental Animal Center of Nanjing Medical University (Nanjing, China). All animal experiments were conducted according to the guidelines approved by the Animal Ethical and Experimental Committee of Nanjing Agriculture University.

### Viruses and vaccination control

The SA14 strain of JEV, which maintained in our laboratory, was propagated in BHK-21 for the plaque reduction neutralization test (PRNT) and challenge test. The viral titers of the supernatants were approximately 6.3 × 10^7^pfu.

MVA (582nd CEF passage), which maintained in our laboratory, was propagated in BHK-21 and its viral titers of the supernatants were approximately 1.0 × 10^8^ pfu.

Recombinant proteins MEP and EDIII(which were used as vaccination control)were expressed in *E. coli* BL21 as previous reported [30], and purified on Ni-affinity chromatography column (Amersham Bioscience HiTrap chelating HP 5mL × 1column) according to the manufacturer’s instructions.

The inactivated JEV vaccine (SA14-14-2 strain, 2.0 × 10^7^pfu) was obtained from ZHONGMU BIO-INDUSTRY CO., LTD.

### Preparation of rMVA-mep

#### Construction of the rMVA-mep

**Figure 1:**
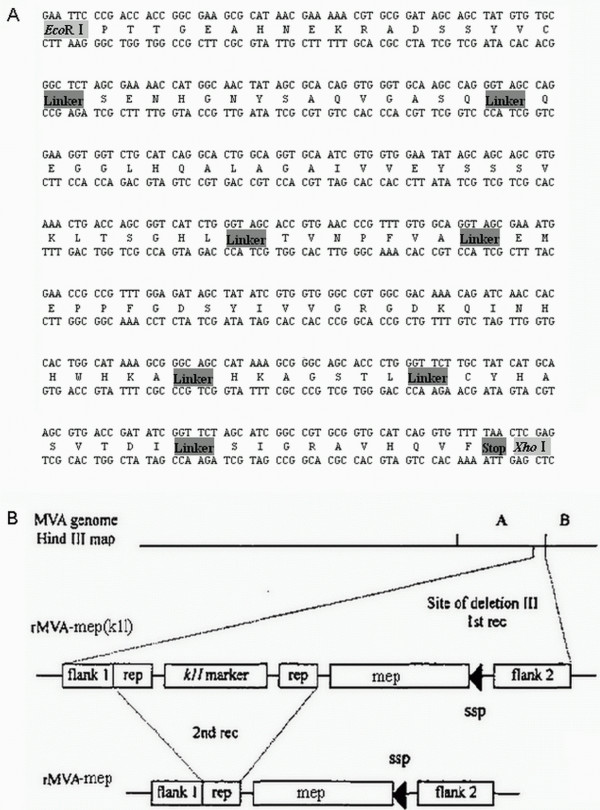
**Construction of the rMVA-mep.A**. Design and construction of the multi-epitope peptide (MEP). The MEP was constructed from six B-cell epitopes and two T-cell epitopes, with a glycine and a serine (GS) as a spacer between epitopes. The amino acid sequences of the epitopes were obtained from the envelope protein of the JEV (AF315119) [29]. **B**. The construction of the rMVA-mep. pGEM-K1L-mep contains the multi-epitope peptide (MEP).

In this paper, according to the previously report [29], the multiple-epitope fragment from the E protein of JEV (SA14-14-2 strain), named MEP (eight epitopes), was designed by arranging the eight epitopes in the order of amino acids (75–92)–(149–163)–(258–285)–(356–362)–(373–399)–(397–403)–(60–68)–(436–445). The amino acid sequence and the nucleotide sequence of MEP are shown in Figure 1A. To minimize interference between adjacent epitopes, each was separated from its neighboring epitope by a glycine and a serine codon [29]. The multiple-epitope gene was chemically synthesized by Invitrogen Biotechnology Co. Ltd. (Shanghai, China) and cloned into the transfer vector pGEM-K1L plasmid and named pGEM-K1L-mep (Figure 1B). 

The MVA recombinants were produced according to the manufacturer’s instructions [31] on BHK-21 cells, named rMVA-mep-BHK-21. Simple, the rMVA-mep-BHK-21, which included rMVA-mep and wild MVA, was purified by serially infecting RK-13 cells, which was called rMVA-mep-RK-13. The rMVA-mep-RK-13 with k1l gene but no MVA was used to transfect BHK-21 cells, in which k1l was removed by intra-genomic homologous recombination. The purified recombinant MVA containing multiple-epitope gene was called rMVA-mep, which was determined by the tissue culture infectious dose 50 (TCID_50_) methods.

#### Identification of rMVA-mep by PCR

To identify that the rMVA-mep contains targeted gene MEP, the genome of RK-13 cells infected with recombinant viruses were prepared, and PCR was used with the specific primers of the targeted gene MEP, and specific gene of wild MVA. Also, the genome of BHK-21 cells infected with recombinant viruses were prepared to detect the host range gene k1l and MEP by PCR method. These primers used were shown in Table 1.

**Table 1:** The primers of Identification of rMVA-mep by PCR

**Gene**	**Primer (from 5’ to 3’)**
Mep	Forward primer: ctcgagatgccgaccaccggcgaagcgca
	Reverse primer: catatgatttttataaaaatttaaaacacctgatgcaccgcacggccgatgctag
K1l	Forward primer: gaatgcacatacataagtaccggcatctctagca
	Reverse primer: caccagcgtctacatgacgagcttccgagtt
MVA (wild)	Forward primer: acataagtaccggcatctctagcacacagc
	Reverse primer: ttcgccggtggtcggcatctcgagagctac

#### Identification of expressed proteins of MEP

Western blot was performed as described previously [29]. The lysates of BHK-21 cells infected with or without rMVA-mep were separated by 15% SDS-PAGE, in which non-infected BHK-21 cells was used as a negative control. The expressions of MEP proteins were identified using JEV-positive serum (PRIONCS, Switzerland) by West-Blotting analysis.

### Mouse immunization

**Figure 2:**
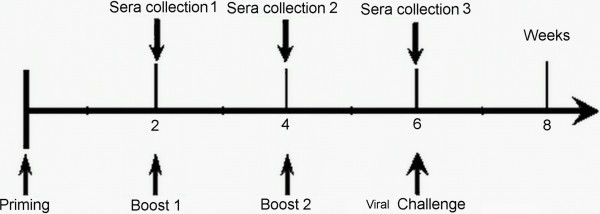
**Vaccination schedule and viral challenge period of in vivo experiment.** Mice were given a first followed by a second immunization and third immunization. The sera samples were collected at 14, 28 and 42 days after immunization. The immunized mice were challenged intraperitoneally with a lethal dose of 5 × 10^6^ pfu of the JEV (SA14 strain). The health of each mouse was assessed twice daily.

Mice were randomly divided into eight experimental groups (fifteen mice each group) and were immunized intraperitoneally (i.p.) on weeks 0, 2 and 4, respectively. The Group 1, Group 2 and Group 3 were given the 10^6^ TCID_50_/0.1mL, 10^7^ TCID_50_/0.1mL and 10^8^TCID_50_/0.1mL rMVA-mep, respectively. Vaccine group mice were each given 0.1ml of JEV (SA14-14-2 strain, 2x10^6^pfu). Also, the recombinant protein immunization groups were given purified 50μg MEP and 50μg EDIII in complete Freund’s adjuvant (CFA) for the first immunizations, in incomplete Freund’s adjuvant (IFA) for the second and the third immunizations. In addition, mice immunized with 0.1mL MVA (2x10^6^pfu) or 0.1mL PBS was used as negative controls (Table 2), respectivley. One week after the final immunization, three mice from each group were sacrificed, and their spleens removed aseptically for in vitro lymphocyte proliferation assay. Also, on 14^th^ day after the third immunization, mice were challenged by i.p. injection of a lethal dose of 5 × 10^6^ pfu of the JEV (SA14 strain) and the survival of the mice was monitored daily up to 15 days post-challenge. The animal groups and immunization doses were described in Table 2, and immunization schedule was shown in Figure 2.

**Table 2:** The diagram of mouse immunization

**Groups**		**Priming**	**Boost 1**	**Boost 2**
rMVA-mep	10^5^TCID_50_	10^6^ TCID_50_/0.1 mL	10^6^ TCID_50_/0.1 mL	10^6^ TCID_50_/0.1 mL
	10^6^ TCID_50_	10^7^ TCID_50_/0.1 mL	10^7^ TCID_50_/0.1 mL	10^7^ TCID_50_/0.1 mL
	10^7^ TCID_50_	10^8^ TCID_50_/0.1 mL	10^8^ TCID_50_/0.1 mL	10^8^ TCID_50_/0.1mL
Inactivated JEV vaccine		0.1 mL (2x10^6^pfu)	0.1 mL(2x10^6^pfu)	0.1 mL(2x10^6^pfu)
EDIII		50 μg CFA	50 μg IFA	50 μg IFA
rMEP		50 μg CFA	50 μg IFA	50 μg IFA
PBS		0.1 mL	0.1 mL	0.1 mL
Wild MVA		0.1 mL(2x10^6^pfu)	0.1 mL(2x10^6^pfu)	0.1 mL(2x10^6^pfu)

*CFA*, complete Freund’s adjuvant; *IFA*, incomplete Freund’s adjuvant.

### Antibody assays

The sera were collected at 14, 28 and 42 days after immunization to perform the antibody assay by ELISA. Simply, the purified multi-epitope protein (rMEP) was coated in 96-well plates (1μg/mL) overnight at 4°C. After blocking, the coated plates were incubated with mouse serum in a sequential dilution of 1:50, 1:100, 1:200, 1:400, 1:800, 1:1600, 1:3200 and 1:6400 for 1.5 h at 37°C. And then, 50μL of a 1:1000 dilution of HRP- conjugated goat anti-mouse IgG, and a 1:2000 dilution of IgG1 and IgG2a were incubated for 45min at 37°C. After extensive washing, tetramethyl benzidine (TMB) substrate was added for 10 minutes at room temperature, and the reaction was stopped with 2M H_2_SO_4_, and absorbance was read at 450 nm. The titer was expressed as the highest serum dilution giving an absorbance > 0.2. The background value was 0.061, and 0.2 was used as cut-off. Each serum titration was assayed in triplicates.

### Estimation of IFN-γ and IL-4 by ELISA

At 7^th^ day after the final immunization, the sera were collected from all experiment groups to measure the productions of IL-4 and IFN-γ using commercially mice cytokine ELISA kits (RD, USA), according to the manufacturer’s instructions.

### Lymphocyte proliferation assay

One week after the final immunization, three mice of each group were killed, and splenocyte were prepared, and were incubated with 10μg/mL rMEP protein in 96-well flat-bottomed microtiter plates (100μL/well, 2 × 10^6^cells/mL in RPMI 1640 medium with 10% FBS). After incubation for 72 h, a final concentration of 20μg/mL MTT (Sigma) was added to each well for 4-h incubation. The medium was removed, and 100μL of DMSO was added into each well [32]. The light absorbance was measured at 570 nm to analyze cell viability expressed as the value of the A570 of cells from immunized mice vs. the control samples. All the data were statistically analyzed with SPSS software.

### Plaque reduction neutralization assay (PRNT)

Neutralization antibodies elicited in immunized mice were valuated by PRNT as described previously [33]. Mouse serum was heat inactivated at 56°C for 30 min. Twofold serial dilutions of murine sera starting at 1:5 were tested, and then incubated with 100 pfu of JEV (virulent SA-14 strain) at 37°C for 1 h. The virus titers were then determined by plaque formation on BHK-21cell monolayers [34,35]. Percentage neutralization was calculated from the number of plaques obtained in the presence and the absence of the serum. The reciprocal of the highest serum dilution giving at least 50% neutralization was regarded as the JEV neutralization titer.

### Statistical analysis

The data were recorded as mean ± standard deviation (SD). Biochemical and physiological parameters were analyzed statistically using one-way analysis of variance (ANOVA) followed by Dunnet-*t*-test using SPSS statistical software to evaluate variations between groups. A value of P < 0.05 was considered to be statistically significant.

## Results

### Generation of the recombinant virus (rMVA-mep) and purification

**Figure 3:**
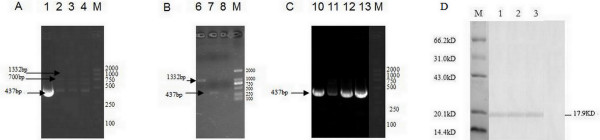
**Identification of rMVA-mep.** (A, B, C) Identification of rMVA-mep by PCR using primers targeted to MEP, to k1l and to MVA gene. (**A**) Lane 1, 2, 3, 4: infected cell focus RK-13-6, RK-13-4, RK-13-3, and RK-13-1, respectively. M: DL2000 marker. (**B**) Lane 6, 7, 8: infected cell focus RK-13-6(k1l gene), RK-13-6(MEP gene), and RK-6(no wild MVA), respectively. M: DL2000 marker. (**C**) Lane 10, 11, 12, 13: infected cell focus BHK-21-6, BHK-21-4, BHK-21-2, and BHK-21-1, respectively. M: DL2000 marker. (**D**) Western Blot analysis of rMVA-mep infected BHK-21. M: Low molecular protein marker; lane 1: Western blot of purified rMEP in *E. coli*. Lane 2: Western blot of purified rMVA-mep infected BHK-21-6 (The sixth generation on BHK-21 cells). Lane 3: Western blot of purified rMVA-mep infected BHK-21-16. Lane 4: BHK-21 cells.

Recombinant viruses carrying wild MVA and host range gene k1l were selected by alternately infecting RK-13 cells and BHK-21cells. The first generation of purified viruses included targeted gene MEP (437bp), specific gene of wild MVA (nearly 700bp) and K1l gene (1332bp) (Figure 3A, lane 4: RK-13-1). To contract with the first generation, the concentration of wild MVA of the fourth generation, RK-13-4 (Figure 3A, lane 2), was obviously lower. There was no wild MVA in the last generation (RK-13-6, Figure 3A, lane 1 and Figure 3B, lane 8), and only the targeted recombinant viruses carrying host range gene k1l (Figure 3B, lane 6) and targeted gene MEP (Figure 3B, lane 7) were presented. Under nonselective growth conditions, k1l was removed by intra-genomic homologous recombination when the recombinant viruses (RK-13-6) were reinfected into BHK-21 cells (Figure 3C). K1l could not be found in the six generation on BHK-21 cells (Figure 3C, lane 10), but not in the fourth, second and first generation (Figure 3C, lane11: BHK-21-4; lane12: BHK-21-2; lane13: BHK-21-1). These results showed that recombinant virus has been purified.

### Western Blotting of rMVA-mep

The rMEP was highly expressed in *E. coli,* and purified with the expected 17.9 kDa protein verified by Western blotting analysis (Figure 3D, lane 1). Moreover, it was observed that the MEP of JEV was stably expressed in BHK-21 cells after rMVA-mep infection, which were proved by Western blotting analysis with the sixth generation of rMVA-mep-infected BHK-21 cells and the sixteenth generation of rMVA-mep-infected BHK-21 cells (Figure 3D, lane 2 and lane 3). No 17.9 kDa protein was found in the negative control of BHK-21 cells (Figure 3D, lane 4). These results demonstrated that the MEP gene was successfully expressed in the rMVA-mep with genetic stability and good immunogenicity.

### Cellular immune responses

**Figure 4:**
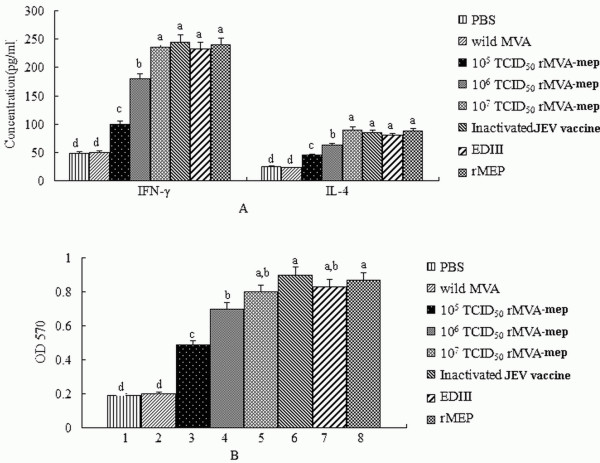
**The cellular-mediated immune responses.** (**A**) The cytokines. After 2^nd^ boost immunized 1 week, the sera from all experiment groups were collected to detect cytokine by ELISA. (**B**) spleen lymphocyte proliferation response. The spleen lymphocytes were incubated in 96-well flat-bottomed microtiter plates and stimulated with rMEP for detecting the spleen lymphocyte proliferation response by MTT. The data are shown as mean ± SD of three independent experiments. Statistically significant differences (P < 0.05) are indicated with different small letters.

Cellular immune responses were evaluated by measuring the production of IFN-γ and IL-4 by splenocytes from mice at 35 dpi when stimulated by rMEP protein. As shown in Figure 4A, IFN-γ and IL-4 productions from mice received rMVA-mep (10^5^TCID_50_, 10^6^TCID_50_, 10^7^TCID_50_), inactivated vaccine, EDIII, and rMEP were significantly higher than that in the PBS and wild MVA (P < 0.05). Mice immunized with rMVA-mep promoted the IFN-γ and IL-4 levels in dose-dependent manner, in which cytokines production of mice immunized with 10^7^TCID_50_ rMVA-mep was observed highest among the three dosages. Also, the levels of IFN-γ and IL-4 were similar among mice immunized with rMVA-mep (10^7^TCID_50_), inactivated vaccine, EDIII, and rMEP. These results suggested that rMVA-mep could induce both Th1 and Th2 type immune response.

The level of lymphocyte proliferation varied among the groups as shown in Figure 4B. The highest occurred in the group immunized with inactivated vaccine of JEV SA14-14-2, and this level was significantly higher than those produced by PBS, wild MVA, or rMVA-mep (10^5^TCID_50_; 10^6^TCID_50_). We also found that the level of splenocyte proliferation observed from the groups immunized with rMVA-mep (10^7^TCID_50_), EDIII, and rMEP was nearly equal; rMVA-mep can enhance antigen-specific splenocyte proliferative response in a dose-independent manner. No significantly lymphocyte proliferation was induced by immunization with PBS and wild MVA.

### Anti-rMEP of JEV antibody titers and subtype analysis

**Figure 5:**
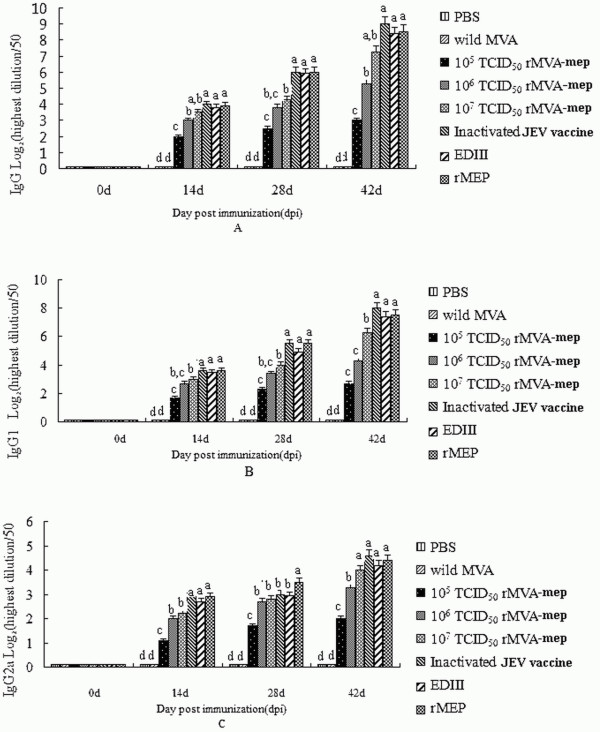
**Specific antibody responses.** The sera were collected on day 14 after prime, 1^st^ boost and 2^nd^ boost immunized for detecting the antibody special to rMEP of JEV by ELISA, respectively. The data are shown as mean ± SD of five mice and are fully representative for the individual mice tested. (**A**) IgG antibody; (**B**) IgG1 antibody; (**C**) Ig2a antibody. Statistically significant differences (*P* < 0.05) are indicated with different small letters.

To estimate the potential roles of rMVA-mep on the humoral immune response, the IgG isotopes profiles of sera from immunized mice at two weeks after three immunizations were measured by ELISA method (Figure 5). These results showed that mice immunized with rMVA-mep generated a significant rMEP-specific antibody response in dose-dependent manner, in which mice injected with 10^7^TCID50 rMVA-mep produced the strongest antibody response among the three reachable dosages (Figure 5A, IgG). Also, it was observed that the level of IgG from mice given the inactivated vaccine, EDIII and MEP were higher than that of mice given with 10^7^TCID50 rMVA-mep. It was found that a combined IgG1/2a response was characteristic of mice immunized using an rMVA-mep prime-boost protocol (Figure 5B, IgG1 and Figure 5C, IgG2a). Moreover, the antibody level of IgG1 was significantly higher than that of IgG2a, which indicated that the IgG1 antibody was the dominant antibody subtype induced by rMVA-mep vaccination. Low levels of both IgG1 and IgG2a were found in control sera.

These results suggested that rMVA-mep might be as potential candidate vaccine for the prevention of JEV infection.

### Neutralizing antibodies

Serum samples collected on days 14, 28 and 42 were further evaluated for their ability to neutralize JEV in vitro by the plaque reduction neutralization assay (PRNT). As shown in Table 3 (2^nd^ boost), although neutralization titer of serum from mice immunized with rMVA-mep (10^5^TCID50 [20 ± 1.33] and 10^6^ TCID50 [33 ± 2.33]) was significantly lower than that of the mice immunized with JEV vaccine (58 ± 2.01), EDIII (56 ± 1.99) and MEP (57 ± 1.99), rMVA-mep vaccination (10^7^TCID_50_ [56 ± 1.01]) almostly produced the equal level of neutralization antibody to that JEV vaccine, EDIII and MEP vaccinations. No JEV-neutralizing antibodies were found in the PBS or wild MVA control group.

**Table 3:** The neutralizing antibody levels and protection against JEV infection in immunized mice

**Treatment**		**PRNT50**^**a**^			**Survival rate (%)**
		**Prime**	**1**^**st**^**boost**	**2**^**nd**^**boost**	
rMVA-mep	10^5^TCID_50_	<5	<8	20±1.33	60^c^
	10^6^ TCID_50_	<7	9±1.33	33±2.33*	85^c^
	10^7^ TCID_50_	<8	18±2.33*	56±1.01**	100^c^
Inactivated JEV vaccine		9±1.33*	29±1.02**	58±2.01**	100^c^
EDIII		8±2.00*	28±1.99**	56±1.99**	100^c^
rMEP		9±1.01*	27±2.01**	57±1.99**	100^c^
Wild MVA^b^		--	--	--	0
PBS^b^		--	--	--	8.33

^a^ Prime-boost-boost vaccinations (days 0, 14 and 28) with rMVA-mep were carried out. On day 14 (prime), day 28 (first boost) and day 42 (second boost) sera were taken and analyzed. 50% plaque reduction neutralizing titer (PRNT50) was shown as the geometrical reciprocal of the sera dilution resulting in a 50% reduction.

^b^ No JEV-neutralizing antibodies were detectable in 1:2 diluted sera of the mice in the PBS and wild MVA groups. n = 12, values represent the mean ± standard error. Standard group is rMVA/M (10^5^ TCID_50_).

* Significance [p< 0.05 to animals immunized with rMVA/M (10^5^TCID_50_)].

**Significance [p<0.01 to animals immunized with rMVA/M (10^5^TCID_50_)].

^c^ Significance (p< 0.05 to 8.33%).

### Mouse protection against JEV challenge

To investigate the development of protective immunity further, all immunized mice were intraperitoneally challenged with a lethal dose (5 × 10^6^pfu) of JEV SA14 at two weeks after the final immunization. After the challenge, mice immunized with rMVA-mep (10^7^TCID_50_), EDIII, and rMEP or the inactivated vaccine showed complete (100%) protection against JEV challenge, but only one mouse (1/12) survived in the PBS control group. Mice of the wild MVA group almost no survived (Table 3).

## Discussion

It was reported that the recombinant multi-epitope peptide (rMEP) from JEV using epitope-based vaccine strategies is capable of inducing remarkable humoral and cellular immune responses and provided complete protection against lethal JEV challenge in mice [29]. Modified vaccinia virus Ankara (MVA) has been used to develop recombinant vaccines against the vital diseases including JEV, with some advantages, including contributing to replication-deficient virus efficiently replicates, high bio-safety, high-level immunogenic heterologous antigens expression. So it has been wildly used to develop genetic engineering vaccines and gene therapy [28]. In this paper, we investigated the potential immune functions of the recombinant multi-epitope peptide (rMEP) from JEV expressed in recombinant MVA as multiple epitope vaccine.

The E protein of JEV is a major immunogenic antigen, which shares various B-cell and T-cell epitopes [36]. Some studies have shown that fusion proteins containing Th epitopes may enhance antibody production against the B cell determinant [37], and the linking of a Th epitope to a CTL determinant is effective in the generation of antiviral CTLs [38]. Moreover, the Th cells activated by a vaccine that contains CD4+ Th epitopes may secrete several CTL-inducing or antiviral cytokines, induce CTL responses, and maintain CTL memory [39]. Ideally, epitope-based vaccines should contain both B-cell and T-cell epitopes (CTL epitopes, Th epitopes) that will serve to induce humoral and cellular immune responses. Therefore, in the present study, the selected epitopes containing six B-cell epitopes {(75–92)–(149–163)–(258–285)–(356–362)–(373–399)–(397–403)}, one CTL epitope (60–68), and one Th epitope (436–445) from the E protein of JEV [29] was constructed and generated the recombinant virus (rMVA-mep), designated rMVA-mep. In this paper, we obtained a recombinant MVA expressing recombinant multi-epitope peptide (rMEP) from JEV with genetic stability and good immunogenicity (Figure 3D).

Balb/c mice are widely used experimental animal models. The immune epitopes and protection functions of JEV are verified on Balb/c mice immunization experiments [29]. Therefore, in this paper, we select the Balb/c mice model to confirm the immune response of multiple linear epitopes (B-cell, CTL and Th) of JEV expressed in recombinant MVA. Although the results of Figures 4 and 5 showed no difference of IFN-γ, IL-4, IgG1, and IgG2a titers among 10^7^ TCID_50_ rMVA-mep, recombinant ED3 and inactivated JE vaccine, it was also found that live rMVA-mep elicited strongly immune responses in dose-dependent manner, and the highest level of immune responses was observed from the groups immunized with 10^7^ TCID_50_ rMVA-mep among the experimental three concentrations. Our paper proved that in the mouse model experiments, live rMVA-mep could elicit strongly humoral and cellular immune responses. Unexpectedly, expect for humoral immune responses, recombinant ED3 and inactivated JE vaccine also could induce cellular immune responses. Additionally, the optimum concentration of live rMVA-mep to trigger the maximum immune responses during the immunization is unidentified, which need to be further verified.

In this paper, it was found that mice immunized with rMVA-mep produced the high level of the antibody production with the major antibody subtype IgG1 and the minor antibody subtype IgG2a. Our data showed that rMVA-mep immunization could trigger the strongly cytokine productions in which the levels of IFN-γ were higher than that of IL-4. It has been reported that IgG1 is believed to indicate a humoral immune response, whereas IgG2a is indicative of a cellular response [40]. Furthermore, cytokines play vital roles on various immune responses. IFN-γ, the representative factor of Th1 type immune response, is produced by stimulated T cells and has important immunomodulatory effects [41]. IL-4 can promote B-cell differentiation and enhance the production of antibodies by sensitized B cells [42], which is the representative factor of Th2 type immune response. These results suggested that rMVA-mep could induce both the humoral and cellular immune responses. It was proved that the rMVA-mep vaccination produced strong antibody response in dose-dependent manner, and a mixed Th1/Th2 response in a mouse model.

These findings are consistent with previous studies that rMEP could induce fine immune responses. Although the levels of the neutralizing antibodies from mice immunized with three concentrations of rMVA-mep were lower than that of inactivated JEV vaccine, EDIII and rMEP controls at the prime and 1^st^ boost immunizations, the level of the neutralizing antibodies from mice immunized with 10^7^TCID_50_ rMVA-mep was almost equal to that of these controls at the 2^nd^ boost immunization. Also, the levels of the neutralizing antibodies from mice immunized with three concentrations of rMVA-mep were increased in dose-dependent manner. It was found that the levels of the neutralizing antibodies from mice immunized with three concentrations of rMVA-mep were increased with three time immunization. Furthermore, it was observed that 10^7^TCID_50_ rMVA-mep vaccination elicited high-level neutralizing antibody and protection against JEV challenge. As for the duration of neutralizing antibodies, it was required to be further investigated. These results suggested that rMVA-mep might be an attractive candidate vaccine for preventing against JEV infection.

In conclusion, we first constructed the recombinant viruses (rMVA-mep) expressing recombinant multi-epitope peptide (rMEP) from JEV. It was demonstrated that rMVA-mep vaccination can elicit both humoral and cellular immune responses in a mouse model, such as antibody and cytokines productions increase, and protection response against JEV challenge. These results suggested recombinant MVA/M might be an attractive candidate vaccine for JEV infection.

## Competing interests

The authors declare that they have no competing interests.

## Authors’ contributions

FJW and XLF carried out most of the experiments and wrote the manuscript. QSZ, HYH, BZ, XDL, RP, JZ, WLD, QTL and PYC helped with the experiments. RBC designed the experiments and revised the manuscript. All of the authors read and approved the final version of this manuscript.
